# The Local Integrity Approach for Urban Contexts: Definition and Vehicular Experimental Assessment

**DOI:** 10.3390/s16020154

**Published:** 2016-01-26

**Authors:** Davide Margaria, Emanuela Falletti

**Affiliations:** Istituto Superiore Mario Boella (ISMB), Via P.C. Boggio 61, Torino 10138, Italy; falletti@ismb.it

**Keywords:** local integrity, local protection levels, prototype, Global Navigation Satellite System, Vehicular Ad hoc Network

## Abstract

A novel cooperative integrity monitoring concept, called “local integrity”, suitable to automotive applications in urban scenarios, is discussed in this paper. The idea is to take advantage of a collaborative Vehicular Ad hoc NETwork (VANET) architecture in order to perform a spatial/temporal characterization of possible degradations of Global Navigation Satellite System (GNSS) signals. Such characterization enables the computation of the so-called “Local Protection Levels”, taking into account local impairments to the received signals. Starting from theoretical concepts, this paper describes the experimental validation by means of a measurement campaign and the real-time implementation of the algorithm on a vehicular prototype. A live demonstration in a real scenario has been successfully carried out, highlighting effectiveness and performance of the proposed approach.

## 1. Introduction

The concept of GNSS *integrity* is conventionally defined as the measure of trust that can be placed in the correctness of the information supplied by a navigation system. This concept originates from the classic integrity framework, which was principally defined in the aviation context in the last decades. Since this framework was intended for *Safety-of-Life* (SoL) applications, it also includes the ability of the system to provide timely warnings to users when the system should not be used for navigation. Extensive studies have been conducted on these aspects and a comprehensive literature is available (e.g., see [1] and references therein).

In recent years the concept of GNSS integrity has started to become of interest not only in the aeronautical field, but also in other transportation sectors, where the availability of a “reliable” Position, Velocity, and Time (PVT) information has become a pressing need. Examples of such newcomers are *safety-critical* or *liability-critical* applications especially in vehicular contexts, where the availability of a Protection Level (PL) associated to PVT data from a GNSS receiver can offer a clear added value (e.g., autonomous driving, law enforcement, Pay as You Drive insurance, and electronic toll collection [2]).

Nonetheless, it has been argued that the applicability of the aviation-born integrity to other transportation fields is far from being straightforward: a deep reconsideration of the approach is necessary in order to effectively exploit it in non-aviation operations [2,3]. In fact, the classic GNSS integrity is known to be affected by conceptual and practical limitations when applied to urban contexts [4,5]:

- Conventional analytical models used to predict the pseudorange error variances (assuming an open-sky satellite visibility) may be inconsistent for mass-market automotive receivers. In fact the models for the User Equivalent Range Error (UERE) are based on line-of-sight propagation with diffuse ground multipath only, and they often assume the availability of differential corrections. They do not account for possible time- and space-varying impairments (e.g., limited satellite visibility, non-line-of-sight signals, fast fading), whose probability of occurrence is high in urban scenarios [2];
- Typical requirements of integrity and continuity risks associated to aviation operations per phase of flight could be too conservative with respect to the requirements of vehicular applications, especially in non-SoL cases [3];
- In the case of *Satellite-Based Augmentation System* (SBAS, for example EGNOS in Europe), the typical availability and applicability of GNSS integrity information can be limited in urban canyons. In detail, either the geostationary satellites can be blocked or the obtained PLs can result too conservative for a non-SoL vehicular application (e.g., as demonstrated by the measurement campaigns reported in [6]);
- The classic integrity approach does not take advantage of collaborative architectures, implying the potential availability of additional information from multiple GNSS receivers connected by Vehicle-to-Vehicle and Vehicle-to-Infrastructure communications (e.g., in a VANET) [5].

These theoretical problems and practical limitations are gaining special attention in both the GNSS and vehicular communities, where the investigation of possible solutions is an active research topic. Some standalone solutions have already been proposed, focusing on autonomous integrity approaches and/or fusion of a GNSS receiver with other sensors available on board of a vehicle (e.g., inertial measurement unit, odometer, vision sensors, *etc.*) [4,5,7,8,9].

An innovative integrity monitoring solution has recently been introduced in [10]. This novel “local integrity” concept is based on a collaborative architecture tailored to the vehicular context and aims at overcoming the limitations of classic integrity. The general idea is to take into account not only the GNSS system, but also the local environment nearby the receiver, focusing on expected signal degradations (e.g., multipath and non-line-of-sight signals). Aggregated position- and time-dependent information can be used in order to estimate an equivalent range error essentially dependent on the local environment around the receiver (local UERE): such data aggregation represents a first important point in order to characterize the local errors in nominal conditions. As a second step, the aggregated data can be shared by means of a VANET infrastructure in order to estimate “Local” Protection Level (LPL) values. Further steps can include the definition of proper integrity checking strategies and related parameters (*i.e.*, testing LPL values *versus* properly identified alarm limits). These strategies are needed in order to detect and possibly mitigate local errors in non-nominal conditions.

In addition, once available, the local UERE information can become fundamental for properly weighting the GNSS receiver output with respect to the position information from other sensors available on-board of a vehicle. This approach can potentially improve the robustness of the combined position shown to the car driver, for example in case of frequent GNSS signal outages.

Concerning the first step for the implementation of a collaborative integrity monitoring, which is the estimation of the local UERE, a preliminary investigation has already been carried out by the authors in [11]. These analyses confirmed the feasibility of local integrity concept, focusing on the repeatability of GNSS degradations in an urban scenario and on how to properly combine the GNSS data for local UERE estimation. A live demonstration of the developed prototype has successfully been carried out in 11 December 2014, during the final event of the GLOVE project [12].

This paper then continues the previous work, analysing new results that prove the suitability of the proposed approach to vehicular applications and demonstrate its performance in a real urban scenario. The real-time prototype implementation of the local integrity algorithm is also presented.

This paper is an expanded version of a previous conference paper [13], where preliminary results have been anticipated. The contents have been extended, also taking into account useful feedbacks and comments from the reviewers and the audience of the conference. Among other improvements, the performance assessment has been enhanced by means of Stanford diagrams in order to highlight the performance and conservativeness of the proposed approach. More details on the implementation of the vehicular prototype have also been provided.

After this introduction, the next section describes the local integrity approach, presenting a suitable architecture and the estimation method for the local UERE. The following sections are focused on the experimental validation of the method and its implementation in a prototype demonstrator. Obtained results from field tests and vehicular data collections are then presented, followed by the final remarks.

## 2. Local GNSS Integrity Approach

### 2.1. General Idea and Suitable Architecture

**Figure 1:**
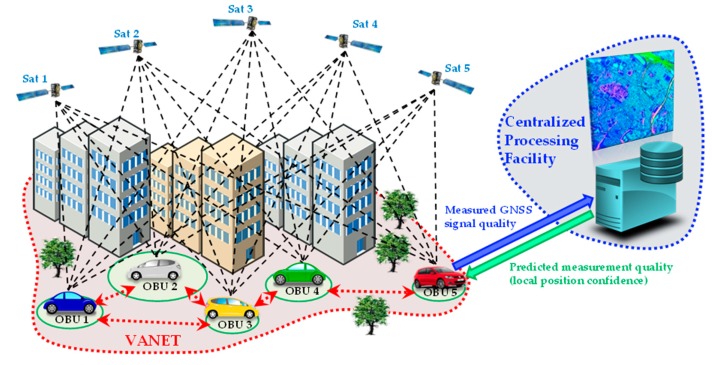
Illustration of the local integrity architecture: VANET communications are exploited for updating a data base with information on local GNSS measurement quality and then for predicting the local position confidence of each vehicle.

The local integrity is based on the concept of using connected vehicles as sensors in order to implement a cooperative monitoring of the integrity of GNSS signals. As shown in Figure 1, the general idea is to take advantage of multiple observations of GNSS signals shared by multiple On-Board Units (OBUs) mounted on vehicles participating to a VANET architecture [10].

It must be pointed out that the possible system architecture is not just limited to a VANET with a centralized processing facility, as in Figure 1. In fact, depending on the user case and also on the available communication links (VANET or 3G/4G, as a backup in case of insufficient coverage), multiple architectures can be considered for the local integrity, also including de-centralized and non-real-time approaches:

- a (fully or partial) post-processing-based approach, in case of insufficient or intermittent connectivity of the vehicles with the centralized processing facility. In this case GNSS measurements are temporarily stored on board of each vehicle. They are downloaded and transmitted to the server only when the vehicle is connected to the network (e.g., once per day or per month). The suitability of this non-real time approach depends on the application requirements (e.g., in case of a road tolling solution);
- a server-side implementation, requiring one-way communications from the vehicles to the control centre, with centralized processing and without on-board data storage and display (e.g., fleet management);
- a vehicle-side real-time implementation, requiring a two-way communication in order to also display local integrity information on-board of each vehicle (useful for example in a collision avoidance system);
- a completely de-centralized approach, based on a collaborative architecture without the need for a control centre (e.g., in case of info-mobility solutions for leisure applications).

However, for the sake of simplicity but without losing generality, a centralized architecture is considered in the following discussion. The vehicles are assumed to travel the same road segments with a comparable velocity and to be equipped with OBUs including GNSS receivers with similar characteristics. This assumption implies a comparable pseudorange measurement quality for receivers in similar operative conditions, or the adoption of proper compensation factors in case of different classes of receivers (e.g., consumer-grade or survey-grade). At this point it is necessary to identify a measurable quantity related to the pseudorange statistics in order to highlight possible local signal degradations.

### 2.2. Exploitation of Pseudorange Residual Measurements

A useful raw source of information for assessing the GNSS signal quality can be identified in the *pseudorange residual measurements* or a function of them [10,11].

Such residuals are conventionally defined as the differences between observations and estimated observations of pseudorange measurements [14]. They can be computed starting from the measured pseudoranges for each satellite and the geometrical ranges between the satellites and the user. The measured pseudoranges can be possibly corrected (e.g., applying SBAS corrections), whereas the geometrical ranges are a-posteriori computed knowing the satellite position and the estimated position of the receiver (linearization point).

In case of a simple Least Squares (LS) solution, the *vector of pseudorange residuals* $w_{n}={\left(w_{1,n}w_{2,n}...w_{N_{sat},n}\right)}^{T}$, referred to a specific discrete time instant (*n*) and a set of *N_sat_* satellite-user pseudoranges, can be computed as [14]:

(1) $$w_{n}=\delta \text{​}y_{n}-H\delta x_{\mathrm{LS},n}$$

where:

- $\delta \text{​}y_{n}={\left(\delta \text{​}y_{1,n}\delta \text{​}y_{2,n}...\delta \text{​}y_{N_{sat},n}\right)}^{T}$ is the vector $R^{N_{sat}\times 1}$ containing the differences between nominal (geometrical) ranges and pseudoranges (observations) measured at the *n*-th time instant for each *i*-th satellite (with *i* ranging from 1 to *N_sat_*);
- H is the linear connection matrix $R^{N_{sat}\times 4}$, derived from the linearization of the PVT solution problem about the selected linearization point;
- $\delta x_{\mathrm{LS},n}={\left(\delta x_{n}\delta y_{n}\delta z_{n}-\delta t_{n}\right)}^{T}$ is a vector $R^{4\times 1}$ containing the position-time deviation obtained after the LS solution at the *n*-th time instant [15].

As noticed in [10,11], various consumer-grade receivers (e.g., uBlox-6T [16]) can directly output the residuals by means of a specific sentence ($GPGRS) defined in the common standard output format NMEA 0183, from the National Marine Electronics Association [17]. For this reason, it is possible to assume that vehicles in a VANET can time-stamp and share their respective residuals together with their estimated positions. All these data can be combined by a central processing facility, thus building a data base in the form of a digital map, with a reasonable granularity. Notably, it is possible to combine GNSS observations taken at the same place over multiple days due to the repeatability of satellite/receiver geometrical conditions, depending on the satellite orbits [10]. In fact, every sidereal day (23 h and 56 min) a GPS satellite passes over the same place on Earth with the same azimuth and elevation and then with comparable signal degradations due to multipath effect. This concept can be extended to GNSS signals other than GPS (e.g., Galileo satellite orbit repeat period is equal to 10 d [18]). In case of a decommissioned (or replaced) satellite, its related data can be reset in the data base.

### 2.3. Effective UERE and Local Protection Levels

The considerations in previous sections can be used in order to estimate the quality of the pseudoranges without resorting to generic analytical UERE models, as in the classic integrity framework. A possible solution is to adopt a novel “*effective UERE*” parameter, which is defined in [10] as the ensemble average, at a certain position and time, of several “instantaneous” estimates of the covariance of the residuals. In synthesis, this parameter can be computed by means of the following operative steps:

1. Collect *M* independent observations of the vector of pseudorange residuals $w_{n}$ (with *n* = 1, …, *M*) from a set of *N_sat_* satellites used in PVT solution. The observations $w_{n}$ are obtained from different closely-spaced receivers in the VANET and/or combining measurements performed in different days, but with nearly the same geometrical conditions, velocity, and then similar signal degradations.
2. Compute the ensemble mean of the covariance matrix of the residuals as:
  (2) $${\bar{\Sigma }}_{w}=\frac{1}{M}\sum_{n=1}^{M}\left(w_{n}\;{w_{n}}^{T}\right)$$
  where it must be noted that $\left(w_{n}\;{w_{n}}^{T}\right)$ is a square matrix $\left(R^{N_{sat}\times N_{sat}}\right)$ representing an instantaneous estimate of the covariance matrix at the *n*-th time instant.
3. Estimate the *effective UERE variance* $\left({\hat{\sigma }}_{\mathrm{UERE},eff}^{2}\right)$, starting from the Frobenius-norm of ${\bar{\Sigma }}_{w}$ (*i.e.*, the square rooted trace of ${\bar{\Sigma }}_{w}^{T}{\bar{\Sigma }}_{w}$) and the number of satellites *N_sat_*.
  (3) $${\hat{\sigma }}_{\mathrm{UERE},eff}^{2}=\frac{{\left\|{\bar{\Sigma }}_{w}\right\|}_{\mathrm{Fro}}}{\sqrt{N_{sat}-4}}=\sqrt{\frac{\sum \mathrm{diag}\left\{{\bar{\Sigma }}_{w}^{T}{\bar{\Sigma }}_{w}\right\}}{N_{sat}-4}}$$
4. Obtain the *effective UERE standard deviation* $\left({\hat{\sigma }}_{\mathrm{UERE},eff}\right)$ as:
  (4) $${\hat{\sigma }}_{\mathrm{UERE},eff}=\sqrt{{\hat{\sigma }}_{\mathrm{UERE},eff}^{2}}$$

Such an aggregated $\left({\hat{\sigma }}_{\mathrm{UERE},eff}\right)$ parameter represents a statistical characterization of the “local” environmental effects close to the receiver position, taking into account the satellite/receiver geometry and the measurement quality.

As conceptually shown in Figure 1, this aggregated position- and time-dependent information can be stored in a centralized data base and then properly re-routed across the VANET in order to predict the GNSS signal quality in each position and at each hour in the GNSS time (orbital) frame.

**Figure 2:**
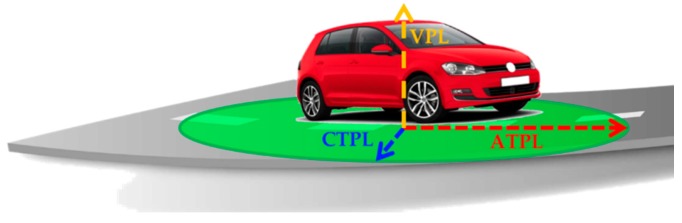
Concept of Along-Track, Cross-Track, and Vertical Protection Levels.

These operations represent the first step in order to estimate LPL values. In detail, LPLs can be defined adopting an Along-Track, Cross-Track, Up (ACU) reference frame, related to the orientation of a car in a road segment, as in Figure 2.

The Along-Track, Cross-Track, and Vertical Protection Levels (ATPL, CTPL, VPL) can be expressed as:

(5) $$<x>\mathrm{PL}=k_{<x>}\cdot {\hat{\sigma }}_{\mathrm{UERE},eff}\sqrt{{\left[{\left(H_{\mathrm{ACU}}^{T}H_{\mathrm{ACU}}\right)}^{-1}\right]}_{\left(jx,jx\right)}}$$

where <*x*> stands for either {AT, CT, V} and *j_x_* = {1, 2, 3} respectively; furthermore, $H_{\mathrm{ACU}}$ is the linear connection matrix computed in the ACU frame (depending on the satellite/user geometry) and $k_{\mathrm{AT}},$ $k_{\mathrm{CT}},$ and $k_{V}$ are three multiplicative dimensionless factors which propagate the local position confidences to a level compatible with proper integrity requirements (these should be related to the intended application). Interested readers can find more details and the complete analytical derivation of previous expressions in [10].

## 3. Experimental Validation and Data Analysis

After the analytical definition of the local integrity approach, the method was experimentally validated. In detail, the effective UERE model was adapted to the selected urban scenario in order to characterize local errors and then to verify the conservativeness of the resulting LPL values.

### 3.1. Measurement Setup and Vehicular Data Collections

**Figure 3:**
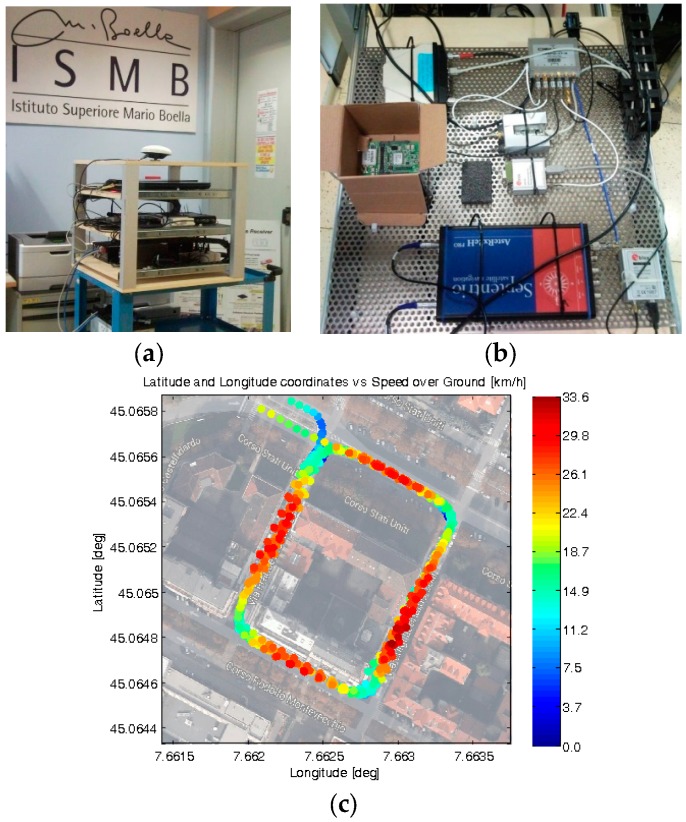
Measurement setup for vehicular data collections: (**a**) Experimental setup; (**b**) Devices under test; (**c**) Example of logged positions and speeds on a selected path, shown in Google Maps^©^.

A GNSS measurement setup, suitable to perform vehicular field tests and data collections, was designed and assembled at ISMB [11]. As shown in Figure 3a,b, this setup includes seven GNSS devices under test. They are from different manufacturers and representative of different receiver categories.

In detail, two survey-grade GNSS receivers were used as references during these tests: AsteRx2eH PRO (Septentrio N.V., Leuven, Belgium) and FlexPackG2-V2-RT2-G (Novatel Inc., Calgary, AB, Canada) and five consumer-grade GPS/EGNOS receivers were compared: LEA-4T (uBlox, Thalwil, Switzerland), uBlox AMY-5M, uBlox EVK-6T, uBlox 6, and IT600 MVK (formerly Fastrax Oy, Espoo, Finland, acquired by uBlox in 2012).

This setup was used for carrying out multiple vehicular measurement campaigns, grabbing pseudorange residuals measurements in an urban area (in Torino, Italy). Figure 3c reports a rectangular path, selected as a preliminary test route. Both the position and speed information logged during multiple consecutive loops by one receiver under test (uBlox EVK-6T [16]) are displayed. Note that the speed of the car in each straight road segment was similar along all passes (reaching a maximum of 33.6 km/h): this fact makes reasonable to combine the outputs of the receivers during successive loops in order to compute an ensemble average.

Preliminary analyses of these measurements were anticipated in [11], where pseudorange residuals were validated as an indicator of local degradations of GNSS signals. In addition, the repeatability and a remarkable spatial/temporal correlation of such measurements were demonstrated in this urban scenario. For this reason, 15 m and 5 min have been identified as reasonable space and time granularities, respectively, for combining these residual measurements (*i.e.*, the resolution for estimating the ${\hat{\sigma }}_{\mathrm{UERE},eff}$ parameter). This result will be used in the next section for building an effective UERE data base and then for extracting a simplified model of local GNSS degradations.

### 3.2. Post-Processing Analyses for Effective UERE Estimation

The feasibility of the local integrity approach in the selected urban scenario was validated by assessing multiple experimental measurements. Ad hoc routines were developed in a MATLAB^®^ environment in order to parse and post-process the NMEA data logs obtained from the receivers under test. The local integrity algorithm was implemented according to Equations (2) to (5), thus including both the ensemble estimate of effective UERE and the computation of instantaneous Local Protection Level values (ATPL, CTPL, VPL).

**Figure 4:**
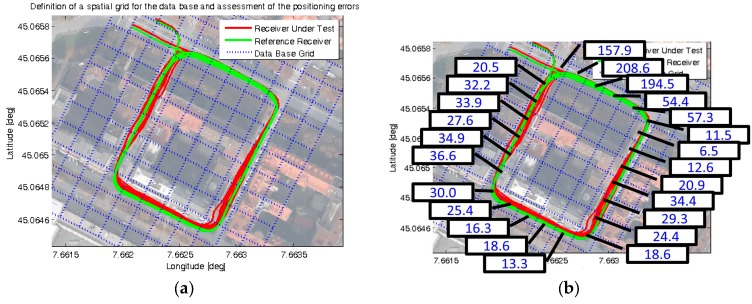
Results of an experimental GNSS data collection: (**a**) Positions logged and spatial grid for combining the measurements, shown in Google Maps^©^; (**b**) obtained values of effective UERE, in meters.

Focusing on the effective UERE estimation, a data base was built from the available observations of pseudorange residuals. An ACU reference frame was adopted, taking into account the local orientation of the road segments with respect to the North direction. A suitable spatial grid was then defined with a resolution of 15 m, as shown in Figure 4a (blue dotted lines). This figure also reports the position information logged during multiple consecutive loops by one receiver under test (red line, obtained from the uBlox EVK-6T [16]) and by a reference receiver (green line, from the Novatel receiver in RTK + IMU mode).

The pseudorange residual measurements collected in a time window of 5 min were separately combined for each cell of the spatial grid, aiming at properly estimating the effective UERE in each location of the selected path. As an example, ${\hat{\sigma }}_{\mathrm{UERE},eff}$ values obtained processing the measurements of the uBlox EVK-6T receiver are reported in Figure 4b. These values were computed for each cell of the path, combining all the observations of residual vectors $w_{n}$ available during the selected 5 min time window from all the satellites used in PVT; the obtained ${\hat{\sigma }}_{\mathrm{UERE},eff}$ values range from 6.5 to 208.6 m, clearly denoting different qualities of the pseudoranges in different locations along this path.

It can happen that different receivers located in a specific cell of the urban grid see dissimilar subsets of the expected satellites in view, due to their slightly different locations and signal reception conditions. For this reason and for the sake of flexibility, instead of directly storing aggregated ${\hat{\sigma }}_{\mathrm{UERE},eff}$ values in the data base, the information about the pseudorange quality has been kept separate for each available satellite. Thus, for each cell of the grid, the averaged covariance matrix of the residuals ${\bar{\Sigma }}_{w}$ was stored as computed from Equation (2), having as diagonal elements the estimated residuals covariance ${\hat{\sigma }}_{w_{j}}^{2}$ for each *j*-th satellite.

### 3.3. Computation of the Local Protection Levels

After building an effective UERE data base as described above (*i.e.*, a different matrix ${\bar{\Sigma }}_{w}$ for each cell of the spatial grid), the ${\hat{\sigma }}_{\mathrm{UERE},eff}$ values were computed as from Equations (3) and (4). The actual number of satellites used by a specific receiver (*N_sat_*) was considered during the computations in order to accordingly reshape the ${\bar{\Sigma }}_{w}$ matrix (*i.e.*, excluding rows and columns related to unused satellites).

The next step for computing the Local Protection Levels, as from Equation (5), is the definition of proper $k_{\mathrm{AT}},$ $k_{\mathrm{CT}},$ and $k_{V}$ multiplicative factors. It must be pointed out that their complete characterization in vehicular cases is an open issue (out of the scope of this work), because they depend on the specific application requirements and on the actual receiver performance. However, in order to validate the feasibility and to simply demonstrate the performance of the local integrity approach, empirical $k_{\mathrm{AT}},$ $k_{\mathrm{CT}},$ and $k_{V}$ factors were set. Such choice aimed at ensuring a sufficient conservativeness of the ATPL, CTPL, and VPL with respect to actual Positioning Error (PE) values. These errors were computed in the Along-Track, Cross-Track and Vertical directions (ATPE, CTPE, VPE) as the difference between a reference track, obtained from a survey-grade receiver (shown as a green line in Figure 4), and the instantaneous positions estimated by the receiver under test (shown as a red line in Figure 4).

An arbitrary success rate of 99% was targeted, intended as the percentage of positive cases where the local protection levels are conservative with respect to the absolute values of the Positioning Errors (in formulas, this condition corresponds to ATPL ≥ |ATPE|, CTPL ≥ |CTPE|, and VPL ≥ |VPE|).

**Figure 5:**
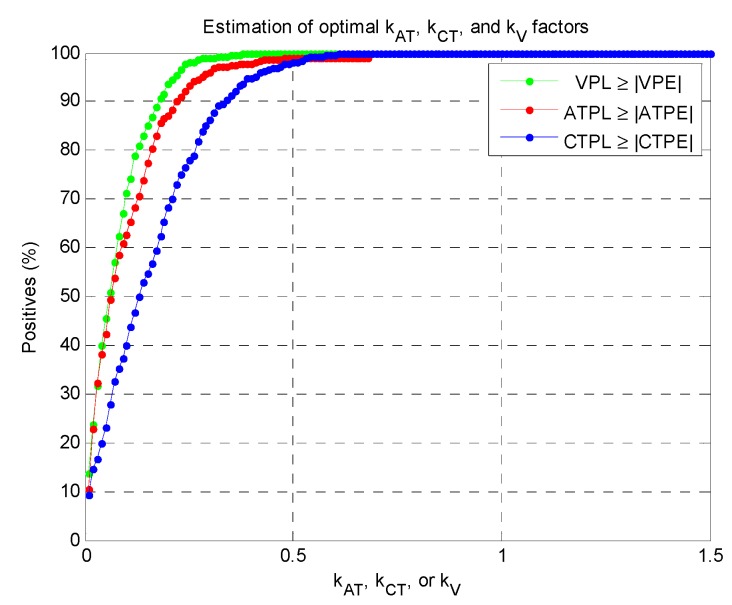
Search for optimal multiplicative factors for the LPL computations.

In this example, most of the errors had magnitude lower than 8 m: focusing on worst-case oriented statistics, the absolute errors assume maximum values equal to 6.8, 8.6 and 8.9 m for |ATPE|, |CTPE| and |VPE|, whereas their 95% quantiles are 3.6, 5.2 and 5.7 m, respectively. In this case the minimum factors ($k_{\mathrm{AT}},$ $k_{\mathrm{CT}},$ or $k_{V}$) sufficient for ensuring at least the 99% of positive cases was 0.6, as demonstrated in Figure 5, with minor differences between the three orthogonal directions.

**Figure 6:**
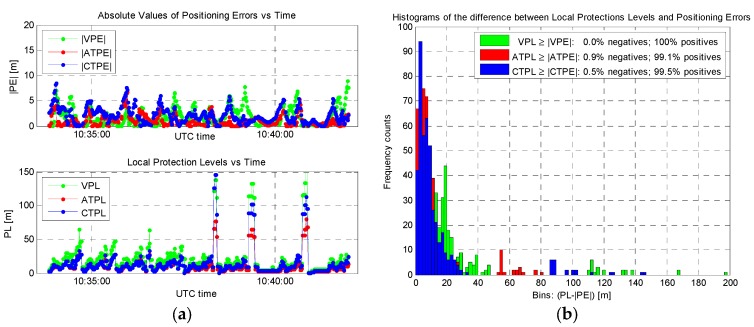
Results of the LPL analysis, assuming *k_AT_* = *k_CT_* = *k_V_* = 0.6: (**a**) Absolute positioning errors *versus* LPL values; (**b**) Conservativeness of LPL values *versus* positioning errors.

At this point, the LPL values were computed choosing for simplicity *k*_AT_ = *k*_CT_ = *k*_V_ = 0.6 and the obtained results are reported in Figure 6, showing instantaneous absolute positioning errors, LPL values, and related statistics. In detail, the maximum LPL values in Figure 6a are equal to 80.3, 145.6 and 198.4 m for the ATPL, CTPL, and VPL, and 95% quantiles equal to 53.3, 84.1 and 109.7 m, respectively. It must be noticed that values larger than 100 m are obtained in three short time intervals only, corresponding to degraded geometrical conditions (*i.e.*, reduced satellite visibility). The conservativeness of these values (especially VPL) is demonstrated in Figure 6b: in fact, non-integer events along the test (*i.e.*, |PE| > PL) represent the 0.0%, 0.9%, and 0.5% of the cases for the Vertical, Along-Track, and Cross-Track directions, respectively.

**Figure 7:**
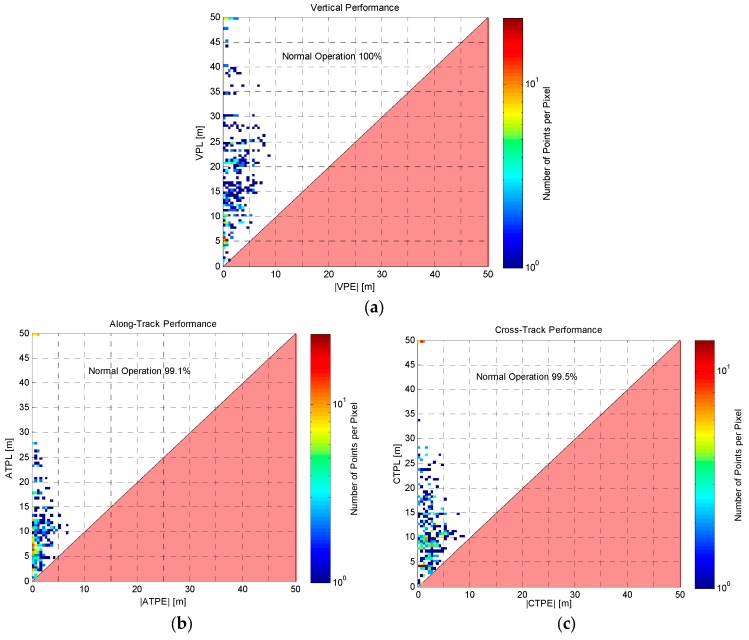
Assessment of the conservativeness of LPL values in terms of Stanford plots: (**a**) Vertical direction; (**b**) Along-Track direction; (**c**) Cross-Track direction.

Aiming to clearly highlight the performance of the proposed approach, Stanford diagrams have also been computed from previous results (by means of MATLAB^®^ scripts adapted from [19]). Figure 7 presents the obtained diagrams for the three orthogonal directions, where it can be noticed that most of the points are located in the upper-left areas of the plots (*i.e.*, corresponding to conservative LPL values with respect to the positioning errors). In detail, the percentages of “Normal Operation” conditions (*i.e.*, 100%, 99.1%, and 99.5% respectively) exactly agree with the percentages of positive cases in Figure 6b: this result provides a further validation of the performance, confirming the conservativeness of the LPL values.

It is worth to remark that previous analyses were carried out on a limited amount of data logged by a single receiver in a small urban area (see Figure 4). The extension to a wider area is possible, but it would require comprehensive data collections and a remarkable computational burden for building the effective UERE data base, which were out of the scope of this work. However, a scenario with a large number of GNSS observations shared by collaborative vehicles could become realistic in the next future, due to the growing penetration of VANET technology and cloud computing facilities for providing new road services.

### 3.4. Simplified UERE Model for Demonstration Purposes

It must be noticed that the described effective UERE data base has applicability limited to a specific urban area and to a specific time slot. Aiming at developing a proof-of-concept demonstrator suitable to a wider urban area, a simplified UERE model was extracted from the available GNSS measurements by means of a curve fitting procedure.

**Figure 8:**
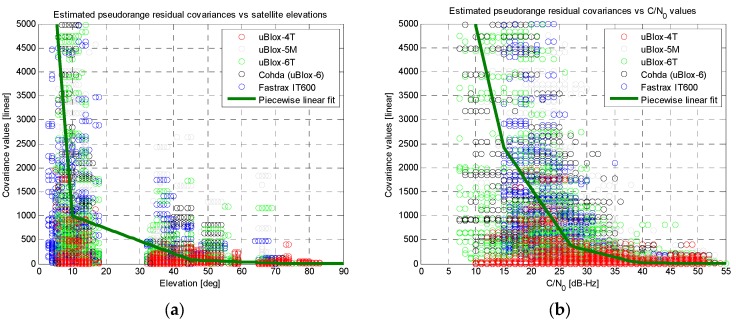
Results of the linear fitting for the covariance of the residuals (linear scale on vertical axes): (**a**) *versus* satellite elevations; (**b**) *versus* C/N_0_ measurements.

The measurements obtained from the five consumer-grade receivers mentioned in Section 3.1 were comparatively assessed. The available pseudorange residuals were analysed *versus* the satellite elevations and *versus* the Carrier-to-Noise density ratio (C/N_0_) values, as reported in Figure 8a,b, respectively.

Quite clear trends can be identified for all the receivers, showing better pseudorange measurement quality (*i.e.*, lower covariance of the residuals) in case of high elevation satellites and high C/N_0_ levels. Similar behaviours can be noticed in Figure 8 for almost all the tested receivers. The only exception is the uBlox-4T, which was the oldest device under test and showed almost flat covariance values *versus* elevation and C/N_0_. Thus, apart this specific device, it is possible to conclude that it is reasonable to build an effective UERE data base by combining measurements from different receivers from same category (consumer-grade), even if they belong to different manufactures. In any case possible calibration factors can be off-line estimated in order to finely tune possible differences between individual devices and/or to compensate for slightly different trends.

**Figure 9:**
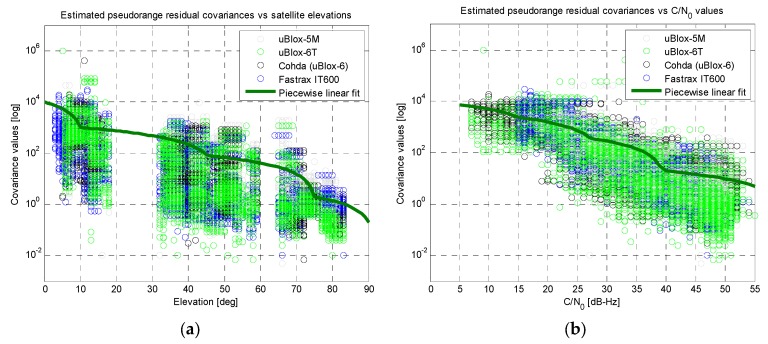
Results of the linear fitting for the covariance of the residuals (logarithmic scale on vertical axes): (**a**) *versus* satellite elevations; (**b**) *versus* C/N_0_ measurements.

Two piecewise linear fits (based on just five points each) were extracted from the trends, as reported by the thicker green lines in Figure 8. Aiming at better highlighting the trends of the covariance values *versus* satellite elevations and C/N_0_ measurements, previous results are plotted with a logarithmic scale for the vertical axis in Figure 9.

In this case the measurements from the uBlox-4T receiver have been excluded, demonstrating a good agreement between the performances of other receivers under test. In addition, the plots in Figure 9 further confirm the validity of the two piecewise linear fits (thicker green lines).

At this point, both the linear fits were used to build a simplified UERE model: starting from the instantaneous elevation and C/N_0_ values for each *j*-th satellite used by a receiver, two separate covariance values can be obtained from the fits in Figure 8a,b, respectively. A weighted average of these two values can be computed and used as an estimate of the covariance of the residuals for each *j*-th satellite (*i.e.*, ${\hat{\sigma }}_{w_{j}}^{2}$). The obtained covariance values have been used as the diagonal elements of an approximated ${\bar{\Sigma }}_{w}$ matrix, setting other off-diagonal elements to zero.

This approach represents a simplified UERE model, which is intended for demonstration purposes only, because the underlying assumptions clearly degrade the reliability of resulting LPL values that must be computed with Equations (3)–(5). Nonetheless, it allows the estimation of effective UERE with a limited computational burden, avoiding the use of a huge data base for storing the covariance matrixes for each grid cell and for each time slot. For this reason, it has been adopted in the prototype implementation presented in next section.

## 4. Proof-of-Concept and Demonstration Results

The demonstrator of the local integrity approach was designed and validated at ISMB premises, as reported in following paragraphs. Such demonstrator was integrated and tested on the vehicular prototype, in collaboration with other partners of the GLOVE consortium [20]. A successful real-time demonstration has been carried out at the final event of GLOVE project [12].

### 4.1. Proof-of-Concept Implementation for Live Demonstration

The local integrity algorithm has been implemented in MATLAB^®^ environment. Ad hoc software modules have been designed and optimized by means of vehicular tests and GNSS data collections on the selected urban scenario. Such modules are capable of parsing and processing in real-time the conventional NMEA output (including $GPGRS sentence) obtained from a consumer-grade GPS/EGNOS receiver (*i.e.*, an uBlox-6 [16]), in order to compute the LPL values.

It must be noticed that these software modules are suitable for processing live data from a local receiver, connected via serial port (USB cable), or from a remote receiver connected via TPC/IP. NMEA data logs can also be processed from file, for offline analysis and performance comparison purposes. In addition, the positioning information, including the LPL values, can be sent via TCP/IP connection to other components in the prototype architecture.

**Figure 10:**
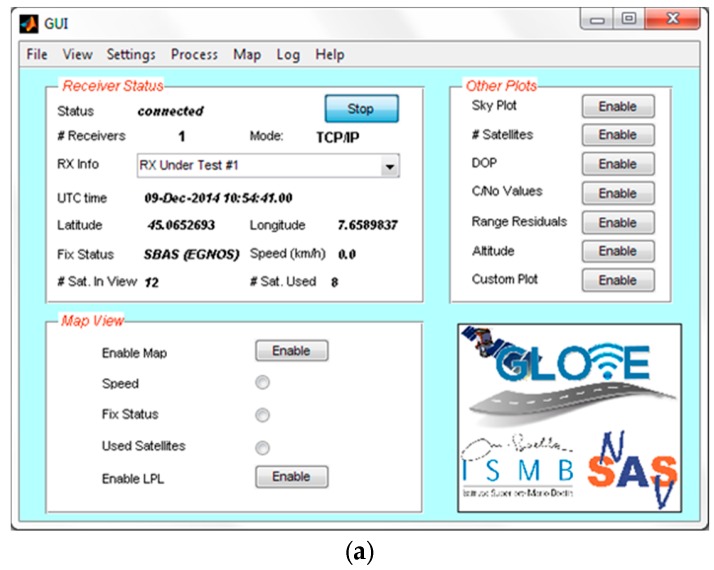

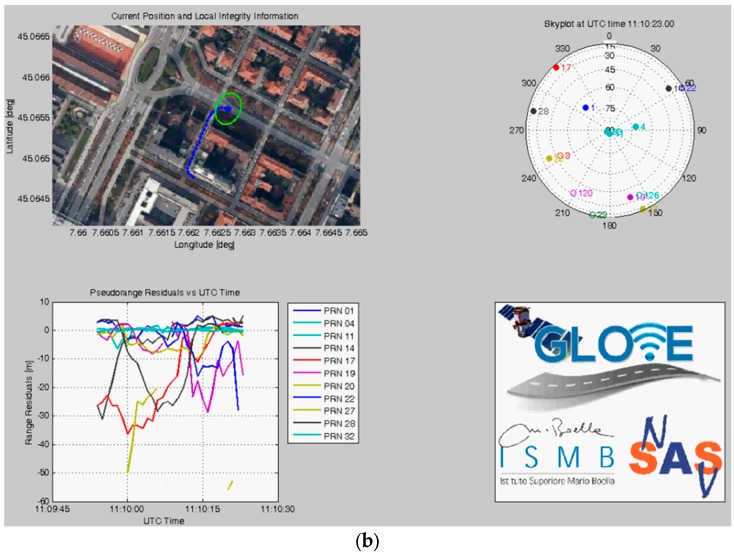
Screenshots of the local integrity software developed in MATLAB^®^ environment for demonstration purposes: (**a**) Graphical User Interface; (**b**) Some results displayed in real-time.

Apart the LPL computation algorithm, a remarkable effort has also been devoted to the development of a proper Graphical User Interface (GUI) for real-time data display. Such GUI, shown in Figure 10, can also be used in order to control the status of the GNSS receiver, log NMEA data from it, and interactively display the local integrity results (*i.e.*, LPL values).

The GUI can also display the receiver outputs (e.g., standalone or SBAS fix status, UTC time, speed, pseudorange residuals, satellite skyplot, C/N_0_ values, *etc.*). In detail, the upper left plot in Figure 10b shows the estimated position of the vehicle on a map, including the latest positions during previous 30 s (blue dots) and the Local Protection Levels, displayed as a horizontal green ellipse around the current position. Such green ellipse provides immediate qualitative and quantitative information about horizontal position confidence. An instantaneous “skyplot” is reported on the upper right plot in Figure 10b, representing the geometry of the satellite constellation as seen by the GNSS receiver. The satellites directly used by the receiver for position computation are denoted with a full circular marker, whereas empty circles represent the satellites in view but not used in the PVT solution.

Finally, the lower left plot in Figure 10b reports the pseudorange residuals for all the satellite signals used by the receiver. It is known that degraded satellite signals usually lead to larger residual values and rapid variations in time. These effects are more likely to occur in case of satellites at low elevation, as for example the satellite with PRN 17, shown in red in Figure 10b.

### 4.2. Prototype Demonstrator of the Local Integrity

**Figure 11:**
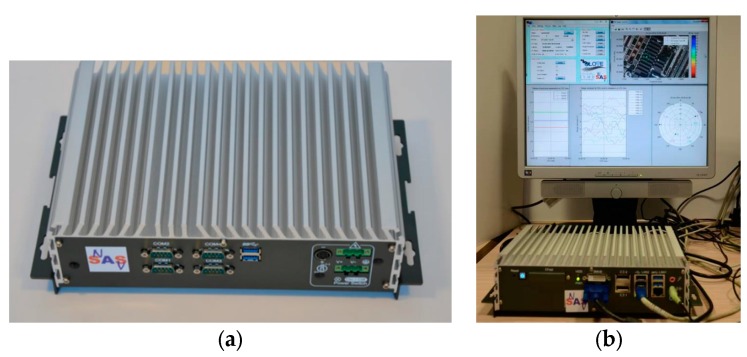
Proof-of-concept demonstrator: (**a**) selected car PC for the demonstrator; (**b**) Car PC properly configured for running the local integrity software and GUI in real-time.

After the software development and testing activities, an automotive-grade computer was configured for running the local integrity software application integrated on board of a demonstration car. Such computer, shown in Figure 11, was selected taking into account specific requirements related to the intended demonstration and the full installation on board of a vehicular prototype enabled for road tests.

**Figure 12:**
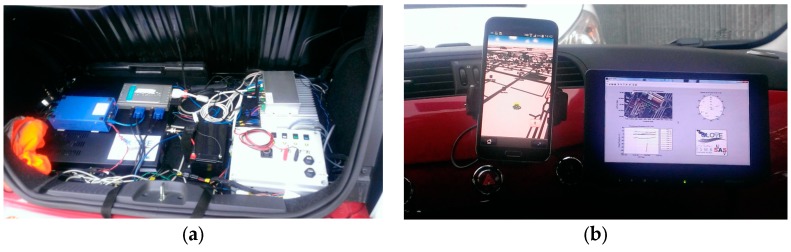
Local integrity demonstrator integrated in the vehicular prototype: (**a**) Car PC mounted in the trunk; (**b**) On-board data display. Reproduced with permission from [12].

The technical specifications of the selected machine (Intel CORE I7-3610QE CPU, 4 GB RAM, 256 GB SSD) were capable to ensure more than enough computational resources for the demonstration purposes. Figure 12 reports two shots of the prototype integrated on board of the demo car.

### 4.3. Obtained Results and Performance Assessment

**Figure 13:**
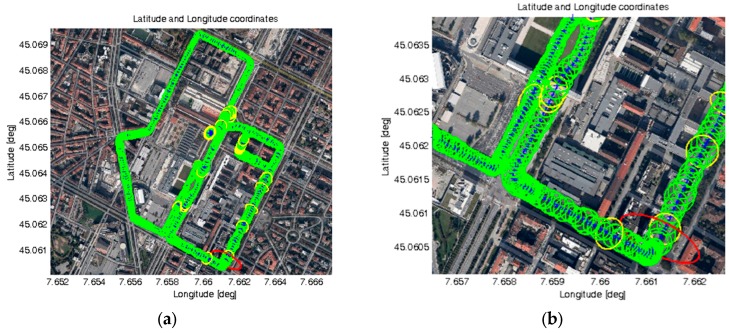

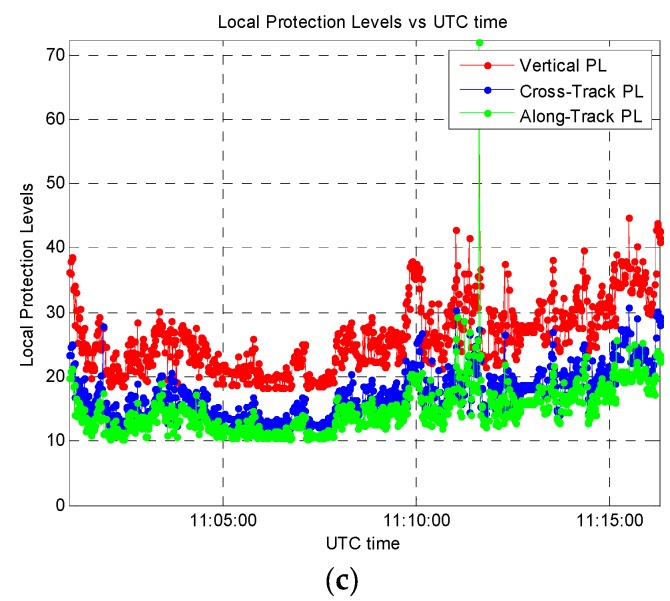
Results obtained on the selected demonstration path: (**a**) Estimated positions and LPL ellipses on the entire path; (**b**) Magnification of a small portion of the path; (**c**) LPL values computed along the entire path *versus* UTC time.

After the completion of the on-board integration, additional vehicular tests have been carried out with the prototype vehicle in order to validate the demonstrator. Figure 13 reports some results obtained during a data collection on the selected path (performed on 5 December 2014).

In detail, Figure 13a shows the full selected path for live demonstration and the obtained LPL ellipses in each location. In this case, three colours have been used for denoting three possible conditions for the maximum value between the semi-major and semi-minor axes of each ellipse (*i.e.*, ATPL and CTPL):

- Green, if such LPL value is below 25 m,
- Yellow, if between 25 and 39 m, and
- Red, if greater than 39 m.

Figure 13a clearly demonstrates that, during the test, most of LPL values were below 25 m (green ellipses). Only a few cases of LPL values above 25 m can be identified (yellow ellipses) and just one case above 39 m (one red ellipse), as shown by the magnification in Figure 13b. Figure 13c reports the LPL values *versus* time. These results have been obtained parsing 900 positions logged during a vehicular data collection lasting 15 min, where the car reached a maximum speed equal to 46.6 km/h. The number of satellites (*N_sat_*) used for computing the position and local PL information ranged from 8 to 11. In detail, as also summarized in Table 1, the following maximum LPL values have been obtained: 72.1, 30.8 and 72.3 m for the ATPL, CTPL, and VPL, respectively, with 95% quantiles equal to 20.9, 24.7 and 36.3 m, respectively.

**Table 1:** Summary of the obtained LPL values on the demonstration path.

Directions	Max. PL Value	95% Quantiles
*Along-Track*	72.1 m	20.9 m
*Cross-Track*	30.8 m	24.7 m
*Vertical*	72.3 m	36.3 m

## 5. Conclusions

A proof-of-concept of the local integrity approach has been presented in this paper, including the theoretical aspects, an experimental validation, and a prototype implementation. Results from a successful live demonstration (carried out during the final event of GLOVE project [12]) have also been reported, showing the achievable performance in a real urban scenario. The proposed approach differs from other state-of-the-art GNSS integrity solutions (e.g., SBAS, GBAS, RAIM) because it performs a collaborative spatial and temporal characterization of local signal degradations. In this way, it does not rely on conventional analytical models for predicting the pseudorange error variances (well assessed in the aviation context only).

The obtained results from this work are promising and worth of deeper investigation and exploitation, especially in the road transport domain. Further research and development activities are needed in order to build a complete integrity framework for vehicular applications. In detail, it is necessary to refine the statistical characterization of different error sources in nominal conditions (*i.e.*, by means of cumulative distribution functions computed on larger data sets and different scenarios) and to adapt/calibrate the local integrity algorithm to different application requirements and operative conditions (e.g., different speed of each vehicle, multiple GNSS signals, and different receiver classes).

Other necessary steps may include the definition of a proper integrity checking strategy, in order to detect and possibly mitigate non-nominal error conditions. In addition, regulatory and standardization aspects are important for the identification and definition of integrity requirements for different road applications, the adoption of a collaborative architecture for sharing GNSS data (e.g., based on VANET), and the standardization of proper communication interfaces.

## Abbreviations

ACU:	Along-Track, Cross-Track, Up
AT:	Along-Track
C/N_0_:	Carrier-to-Noise density ratio
CC-BY:	Creative Commons by Attribution
CT:	Cross-Track
EGNOS:	European Geostationary Navigation Overlay Service
GBAS:	Ground-Based Augmentation System
GLOVE:	joint GaliLeo Optimization and VANET Enhancement
GNSS:	Global Navigation Satellite System
GPS:	Global Positioning System
GUI:	Graphical User Interface
IMU:	Inertial Measurement Unit
ISMB:	Istituto Superiore Mario Boella
LPL:	Local Protection Level
LS:	Least Squares
MDPI:	Multidisciplinary Digital Publishing Institute
NMEA:	National Marine Electronics Association
OBU:	On-Board Unit
PE:	Positioning Error
PL:	Protection Level
PVT:	Position, Velocity, and Time
RAIM:	Receiver Autonomous Integrity Monitoring
RTK:	Real Time Kinematic
SBAS:	Satellite-Based Augmentation System
SoL:	Safety-of-Life
UERE:	User Equivalent Range Error
USB:	Universal Serial Bus
V:	Vertical
VANET:	Vehicular Ad hoc NETwork
